# Dihydropyrimidine dehydrogenase (DPD) polymorphisms knocking on the door

**DOI:** 10.3332/ecancer.2022.1344

**Published:** 2022-01-17

**Authors:** Mauro Daniel Spina Donadio, Dirce Maria Carraro, Giovana Tardin Torrezan, Celso Abdon Lopes de Mello

**Affiliations:** AC Camargo Cancer Center, R Prof Antonio Prudente 211, Liberdade, São Paulo, SP 01509010, Brazil; a https://orcid.org/0000-0002-4705-4802

**Keywords:** fluoropyrimidine, polymorphisms, dihydropyrimidine dehydrogenase

## Abstract

Identifying polymorphisms in the dihydropyrimidine dehydrogenase (DPYD) genes is gaining importance as predictors of fluoropyrimidine-associated toxicity. The recommendation of dose adjustment for chemotherapy guided by the presence of polymorphisms of the *DPYD* gene can potentially improve treatment safety for a large number of patients, saving lives, avoiding complications and reducing health care costs. This article discusses how personalisation of fluoropyrimidine treatment based on the identification of DPYD variants can mitigate toxicities and be cost effective.

## Background

Fluoropyrimidines are one of the most widely used chemotherapy drugs against solid cancers, either as monotherapy or in combination therapy, and more than 2 million cancer patients are exposed annually to this drug, which includes 5-fluorouracil (5-FU) and its oral pro-drugs capecitabine and tegafur [1]. Like all other chemotherapy drugs, fluoropyrimidines also cause toxicities. Adverse drug reactions are a major clinical problem during chemotherapy treatment and often require dose reduction and even treatment interruption. Unfortunately, 10%–30% of patients treated with fluoropyrimidines experience severe or potentially fatal treatment-related toxicity and in 0.5%–1% of these patients the toxicity is lethal [2, 3]. The main adverse events caused by fluoropyrimidines are haematological (leukopenia including febrile neutropenia, anaemia and thrombocytopenia), gastrointestinal (mucositis, stomatitis, diarrhoea, nausea and vomiting) and dermatological (hand-foot syndrome, hair loss and dry skin) but most of these events are mild, reversible and controlled with support measures [4].

In patients with certain enzyme deficiencies that act on the fluoropyrimidine metabolism, however, the use of these chemotherapeutic agents can lead to life-threatening complications, including severe nausea, vomiting and diarrhoea with volume depletion, extensive skin and mucositis changes, pancytopenia with risk of bleeding and infection, cardiotoxicity and neurological abnormalities such as cerebellar ataxia, cognitive dysfunction and altered level of consciousness [5–13]. In these cases, toxicity can occur early during the first treatment cycle, reinforcing the importance of detecting these enzyme deficiencies before the start of therapy, so that personalised dose adjustments of fluoropyrimidine, or even alternative drugs, can be prescribed [14].

The fluoropyrimidine toxicity involves a complex and multi-step mechanism responsible for the drug and its products metabolism and excretion. One of the main steps in the cascade of 5-FU metabolism involves the dihydropyrimidine dehydrogenase (DPD) enzyme, coded by the* DPYD* gene. The genetic factor is the main factor responsible for this enzyme activity and polymorphisms can eventually modify drug metabolism, resulting in drug accumulation and toxicity. Interindividual genetic variation in certain genes is responsible for a significant proportion of adverse reactions and can identify biomarkers that are predictive of the risk of toxicity associated with fluoropyrimidine [15, 16]. Identifying these variants, then, is a relevant effort because it has the potential to greatly improve the safety of a large number of patients.

We conducted a critical review on the mechanisms of fluoropyrimidine toxicity focusing on new molecular findings and recommendations. Moreover, we explored the burden of DPD testing in a developing country such as Brazil.

### Fluoropyrimidines and metabolism pathways

The main fluoropyrimidine is 5-FU, an antimetabolite chemotherapeutic agent that was developed in 1957 by Heidelberger [17]. This drug is widely used in many neoplasms and is a cornerstone treatment for gastrointestinal malignancies. 5-FU is a prodrug that requires intracellular conversion to cytotoxic metabolites with antitumour effects. Of the entire dose administered, the majority is degraded by the catabolic pathway (about 80%), another part is directly excreted in the urine and only 1%–3% of the 5-FU is anabolised to cytotoxic metabolites [18–21].

In the anabolic pathway, 5-FU is metabolised in tissues to 5-fluoro-2′-deoxyuridine and then to 5-fluoro-2′-deoxyuridine-5ine-monophosphate, the active metabolite of the drug. The active metabolite inhibits the enzyme thymidylate synthase, resulting in inhibition of DNA synthesis and repair, inducing cell apoptosis. In addition, the toxic effects resulting from the partial incorporation of 5-FU and its metabolites in DNA and RNA contribute to the mechanism of action of the drug [22, 23]. If there is reduced activity of the enzymes involved in the catabolism of 5-FU, the result can be a substantial increase in the drug’s half-life and, therefore, an increased risk of severe toxicity [18–21].

The second most used fluoropyrimidine is Capecitabine that is metabolised to 5-FU in three consecutive steps, first metabolised to 5′-deoxy-5-fluorocytidine by carboxylesterase, which is subsequently converted to 5′-deoxy-5-fluorouridine by cytidine deaminase and finally to 5-FU by thymidine phosphorylase enzyme. Tegafur, in turn, is metabolised to 5-FU and to the less cytotoxic metabolites 3-hydroxytegafur, 4-hydroxytegafur and dihydrotegafur by Cytochrome P450 2A6 (CYP2A6) [22, 23].

### Genetic variants and their impact on fluoropyrimidines

Genetic variants in the genes coding the metabolic pathway enzymes can alter the metabolism of 5-FU and are clinically significant predictors of fluoropyrimidine toxicity: genetic polymorphisms of the *TYMS* gene (responsible for the enzyme thymidylate synthase) and the enzyme methylenetetrahydrofolate reductase gene are described, although rare. In addition, the variation in cytidine deaminase (*CDA*) expression was associated with polymorphism in the *CDA* promoter region, with an impact on gemcitabine and capecitabine metabolism [15].

The most well-known genetic variant in this scenario is the deficiency of the 5-FU metabolic enzyme, DPD. In 39%–61% of patients with severe toxicity to chemotherapy, the reduced activity in the peripheral blood mononuclear cells of this enzyme has been found [16].

Variants in *DPYD*, the gene that encodes DPD, are gaining importance as predictors of fluoropyrimidine-associated toxicity because the serum tests that detect them are increasingly available and, based on them, dose adaptation is now recommended by some guidelines, such as the Clinical Pharmacogenetics Implementation Consortium [24], Dutch Pharmacogenetics Working Group [25], already endorsed by the European Association of Clinical and Therapeutic Pharmacology and the European Association of Hospital Pharmacists [25, 26]. In 2020, the European Medicines Agency recommended preventive testing for *DPYD* variants before starting cancer treatment with 5-FU, capecitabine and tegafur [27]. This recommendation, however, has not yet been endorsed by the Food and Drug Administration (FDA), European Society of Medical Oncology or the National Comprehensive Cancer Network.

In the catabolic pathway, DPD is the first enzyme that acts by converting 5-FU into dihydrouracil (FUH2) and, although the enzyme has been shown to be present in several tissues, it is believed that the liver is the main organ responsible for 5-FU catabolism. After this conversion, the FUH2 is subsequently metabolised to its final metabolite 5-fluoro-β-alanine, which is excreted in the urine[4]. Next to converting 5-FU, the DPD enzyme also converts its endogenous substrate uracil (U) into dihydrouracil (DHU). The pretreatment ratio of serum DHU/U concentrations was investigated as a phenotypic measure of DPD activity. However, the clinical applicability of the DHU/U ratio has been limited by the lack of robust evidence on clinical validity [4, 16].

There are some possible methods to evaluate DPD function and verifying DPD activity: measuring DPD enzyme activity in peripheral blood mononuclear cells; the 2-^13^C-uracil breath test (where ^13^C02 is measured, which is a product of the degradation of 2-^13^C-uracil by DPD and other enzymes involved in the catabolic route of pyrimidines), the quantification of the DHU/U ratio in plasma and measuring the metabolism of a single dose of uracil [23]. However, all DPD phenotyping tests have their limitations and measuring DPD activity in advance on a routine basis is technically and logistically challenging, laborious and expensive [23].

*DPYD* is a highly polymorphic gene, located on chromosome 1p22, with a single copy of 950 kb that covers 23 exons and more than 7,600 genetic variants have been recorded. Although the majority of these variants are intronic variants that can be considered silent, part of this genetic variation is considered responsible for the great variability in DPD activity that is observed in the general population [4, 23].

In fact, several of the investigated variants have been reported to be associated with reduced enzyme activity and have been proposed as potentially associated with severe 5-FU toxicity, but of these variants, only four were consistently associated with a marked decrease in DPD activity and increased toxicity of fluoropyrimidine, with ≥3 grade toxicity according to the National Cancer Institute Common Terminology Criteria for Adverse Events (NCI CTCAE) related to 5-FU in case–control studies [4]. These variants include DPYD*2A single nucleotide polymorphism (SNP) (c.1905+ 1G>A), DPYD*13 SNP (c.1679T>G), SNP c.2846A>T and a collection of SNPs called HapB3 (a new haplotype – hapB3, composed of some variants, such as: c.483+18G>A; c.680+139G>A; c.959-51T>G; c.1236G>A and the likely causal c.1129-5923C>G intronic variant) [16, 28–30].

The initial screening for the most well-known variant, c.1905+1G>A (previously called IVS14+1G>A or DPD*2A), and dose individualisation in patients with this allele has already been shown to improve treatment safety, avoiding fluoropyrimidine associated severe and potentially fatal toxicity [16, 24]. This variant is the most studied in the context of 5-FU toxicity and the first studies suggested that it would be responsible for up to 29% of all toxicities of grade ≥ 3 but, despite recognising that patients with this variant are at increased risk of severe 5-FU toxicity, the proportion of toxicity cases that could be explained by its presence varies widely. In the largest cohort of more than 680 patients treated with 5-FU monotherapy, 5.5% of 5-FU toxicity cases were explained by c.1905+1G>A [31].

Current data suggest that these variants combined are an important contributing factor for the occurrence of adverse events, accounting for at least 20% of the observed cases of severe toxicities related to 5-FU [4].

The DPD phenotype is assigned using a gene activity score (AS) based on the *DPYD* allele functionality (as shown in Table 1) and calculated as the sum of the two *DPYD* variants with the lowest variant activity value [32]. Table 2 contains the main examples of diplotypes present in available commercial tests with the respective AS and their impact on the DPD metaboliser activity.

### The difficulties of analysing individual variants

In addition to a relative consistency between studies in the proportion of toxicity cases that can be explained by the sum of multiple variants of *DPYD*, given a comprehensive genetic screening of the gene, the importance of individual variants was more variable between studies. There are several potential explanations for these variable results in relation to relatively rare individual variants, well discussed by Amstutz *et al* [4]:

- **Population frequency differences:** a small allele frequency difference for a rare deleterious allele in different populations can lead to large carrier frequency differences and accentuate their relative importance for 5-FU toxicity.
- **Sampling effects:** because the *DPYD* variants have such low frequencies, it would be necessary to evaluate a very high number of toxicity events to arrive at a reliable estimate of the importance of a specific variant for a serious adverse event. Still, this can vary with each individual variant. To mitigate this, the ideal approach would be to combine information from multiple variants with comprehensive genetic screening.
- **Therapy-related heterogeneity:** a considerable source of inconsistencies in the results of different studies related to 5-FU toxicity in DPD deficiency is treatment-related heterogeneity as the functional relevance of *DPYD* variation may vary between different treatment regimens and doses of 5-FU. In addition, there is an overlap of toxicities between chemotherapeutic agents, which can increase the risk of adverse effects, as well as drug interactions that directly affect the metabolism of 5-FU, modifying the risk profile for *DPYD* variants. Another aspect is the sequencing of therapy on DPD function, for example, prior use of gemcitabine can induce liver tissue damage and severe toxicity with capecitabine even in the absence of DPD dysfunction.
- **Heterogeneity in toxicity assessment:** another source of inconsistency in the results of different studies related to 5-FU toxicity in DPD deficiency is the form of assessment of 5-FU toxicity. In addition to using different grading criteria for adverse effects, some studies evaluated toxicities at different times during treatments, that is, severe toxicity was not always characterised according to NCI CTCAE grade 3 to 5 in early chemotherapy cycles.

The frequency of the various *DPYD* variants and the associated phenotypes appears to vary significantly between ethnic groups. Considering all four main variants combined, 5%–7% of the white population has a partial deficiency and 0.1%–0.2% has a complete deficiency of the DPD enzyme. On the other hand, about 8% of the African American population has partial DPD deficiency [23, 33]. The Brazilian population is constituted by nearly 500 years of admixture between Africans, Europeans, Native Americans and Japanese enabling peculiar genetic combinations. The allelic frequency of the four main variants according to the Online Archive of Brazilian Mutations (ABraOM) repository, which contains genomic variants identified by whole-exome and whole-genome sequencing from 1.171 unrelated elderly healthy individuals from São Paulo-Brazil, is shown in Table 3 [34]. As Brazil is a large country with great ethnic diversity, *DPYD* allele frequencies are not homogeneous across its subpopulations and studies with specific subpopulations show different allele prevalence. In example, data from 146 individuals from three Amazonian Amerindian populations showed minor allele frequencies of 1% and 2% for DPYD*2A and DPYD*13, and in healthy Brazilians of predominantly African ancestry or self-reported as black the c.557A>G variant was detected at a frequency of 2.6% [35, 36]. For further analysis and discussions in the text, data from the ABraOM repository will be used as a parameter.

### Dose adjustment recommendation guidelines

Patients with low DPD activity are expected to have an increased risk of developing severe or even lethal toxicity when treated with standard doses of 5-FU or capecitabine [23]. Predicted DPD activity can be expressed as the *DPYD* gene AS, which ranges from 0 (none or practically no DPD enzyme activity) to 2 (normal DPD enzyme activity due to homozygosity for fully functional alleles, both attributed to an AS 1). The gene AS is a sum of the two activities of the protein isoforms expressed in both alleles [23, 33]. Carriers of two no function variants (AS 0) or one decreased function variant (AS 0.5) are classified as *DPYD* poor metabolisers; carriers of two decreased function variants or carriers of only one no function variant (AS 1) or carriers of only one decreased function variant (AS 1.5) are considered *DPYD* intermediate metabolisers, and those with only normal function variants are classified as *DPYD* normal metabolisers (AS 2). Each decreased or no function variant is considered to be on a different gene copy and patients may carry multiple normal function variants. As an individual only carries a maximum of two fully functional *DPYD* copies, common normal function variants may be located on the same gene copy as other normal function variants or decreased or no function variants [32].

The guidelines that address the topic, in summary, suggest that individuals with a gene AS of 0 or 0.5 are recommended to avoid 5-FU, capecitabine or tegafur; individuals with a genetic AS of 1 or 1.5 are recommended to initiate therapy with at least 50% of the standard dose of 5-FU or capecitabine but avoid tegafur. A gene AS of 2 (reference value) does not result in a recommendation for dose adjustment for 5-FU, capecitabine or tegafur [23, 33].

Therefore, high-risk patients with *DPYD* risk alleles could receive modified doses of 5-FU or monotherapy as an alternative treatment option with a potentially increased survival benefit compared to a complete discontinuation of 5-FU therapy [4]. Table 4 shows the correlation between genotype, phenotype, DPD AS and respective risk of severe toxicity associated with fluoropyrimidine.

Although the combination of 5-FU with newer cytotoxic agents, for example, the third-generation platinum derivative oxaliplatin or the topoisomerase I inhibitor irinotecan, or targeted therapies such as bevacizumab, cetuximab or panitumumab, resulted in rates of response significantly improved, the effectiveness of the same agents without the 5-FU combination was limited [37].

For homozygous patients carriers of two identical non-functional alleles and compound heterozygous patients carriers of two different non-functional alleles, it is necessary to use alternative agents. The quinazoline folate analogue raltitrexed, which is a thymidylate synthase inhibitor, may be a useful substitute for 5-FU in patients with DPD deficiency, but it is not widely available [38]. Other reported strategies include use of trifluridine-tipiracil (TAS-102) instead of fluoropyrimidine or fluoropyrimidine micro-dosing [39, 40].

### Supportive treatment after severe toxicity associated with DPD deficiency

Most cases of DPD deficiency are diagnosed only after a severe reaction to 5-FU. The management of these patients should include aggressive haemodynamic support, parenteral nutrition, antibiotics, granulocyte colony-stimulating factors (G-CSF) and, when available, uridine triacetate (UT). UT is a specific pharmacological antidote for fluoropyrimidines, an orally administered uridine prodrug approved by the FDA for emergency use after an overdose of 5-FU or capecitabine. It must be administered within 96 hours after the end of the administration of these chemotherapeutic agents. The recommended dose is 10 g orally every 6 hours, making a total of 20 doses. Despite its approval, UT has a high cost and is not commercially available [41, 42].

This supportive treatment for patients with DPD deficiency presenting severe 5-FU toxicity is based only on case reports and the ideal management still lacks evidence. The use and timing of G-CSF, for example, needs to be better assessed and discussed. Studies of preclinical models that do not involve DPD-related toxicity suggest that G-CSF should not be used early [43]. In individuals with higher and sustained serum levels of cytotoxic agents, as in DPD deficiency, the use of early G-CSF may actually be counterproductive and, to assess the best time of use, it may be necessary to dose the serum level of uracil, for example.

### Cost-effectiveness of routine screening

The costs of prospectively carrying out the *DPYD* gene polymorphism tests appear to be effective. In fact, they would save a greater expenditure on supportive care although it is not possible to price preventable death.

A cost-effectiveness study by an Irish institution evaluated 134 patients who started chemotherapy with first-line fluoropyrimidine over 3 years. Thirty (23%) patients developed grade 3/4 toxicity. Of these, 17% revealed heterozygous *DPYD* deleterious alleles. The cost of hospitalisation for patients with a *DPYD* variant was € 232,061, while prospective testing of all 134 patients would have cost € 23,718. This study suggests that prospective tests would result in cost savings because the cost of hospital admission for severe chemotherapy-related toxicity is significantly higher than the cost of prospective *DPYD* testing for each patient starting fluoropyrimidine chemotherapy [44].

The discussion of cost effectiveness in this scenario is very pertinent since almost half a million patients in Brazil and more than 900,000 in South America have cancers that can be exposed to fluoropyrimidine at some point in the treatment of their disease (Table 5). Testing for DPD deficiency in these emerging countries is certainly a factor that impacts the cost of health care, often prohibitive. In Brazil, a single initiative in the public health system setting has been recently published [45]. However, considering the scenario of limited financial resources that these countries present, unfortunately we cannot envision the universal use of genetic tests in the short term.

It may be necessary to find a niche for patients at higher risk for having DPD deficiency or who would be more vulnerable to complications from chemo toxicities, such as morbid and elderly people, and prioritise testing for these groups. We also have to think about whether the group of patients who will receive higher doses of fluoropyrimidine should be prioritised. The risk of toxicity will always be greater with the use of regimens that use, for example, doses of infusional 5FU of 2,400 mg/m² plus 400 mg/m² in boluses, as in the use of 5-Fluorouracil, Leucovorin, Irinotecan and Oxaliplatin (FOLFIRINOX) for pancreatic cancer [46, 47], when compared with the Cyclophosphamide, Methotrexate and Fluorouracil (CMF) scheme that uses a dose of 600 mg/m², as in breast cancer [48]. There are no studies, however, that address the risk of toxicity by associating DPD deficiency and fluoropyrimidine dose. And this is just another unanswered question that will be increasingly asked in the care routine of oncology services.

**Figure 1.**
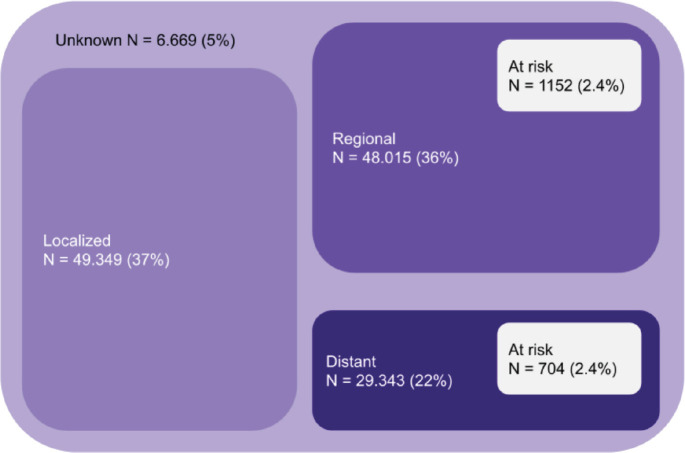
Absolute number of patients at risk considering only colorectal cancer in Brazil (patients at risk considering the sum of MAF (ABraOM)).

As an example, Figure 1 shows the number of patients with colorectal cancer in Brazil according to stage, considering the prevalence according to Globocan, the sum of heterozygotes according to ABraOM, which implies a risk for up to 2.4% of the population, and the proportion per stage of the Surveillance, Epidemiology, and End Results database [52]. Considering colorectal cancer alone, from 48,015 patients with regional disease and 29,343 with distant metastasis, 1,152 and 704 patients would present a risk allele, respectively. It means that more than 1,800 colorectal cancer patients would be at risk of severe toxicity with the use of fluoropyrimidine in Brazil. Despite being an emerging country in which testing for pharmacogenetic variants can economically impact health care, the occurrence of serious toxicities in these patients would certainly have a greater economic impact, with great potential to lead to important morbidity and even death. Although the cost of these consequences cannot be precisely measured, they are potentially preventable if the right measures are taken after mutations are detected. Therefore, more research is needed to understand the cost effectiveness of *DPYD* screening in the setting of low- and middle-income countries such as Brazil.

## Conclusion

Comprehensive genetic testing of *DPYD* is needed in future studies involving the use of fluoropyrimidines. The recommendation of chemotherapy dose adjustment guided by the presence of *DPYD* polymorphisms can become mandatory in the near future due to the potential number of lives that can be saved, complications that can be avoided and costs that can be reduced worldwide. The *DPYD* genotyping and its applicability demand an urgent discussion regarding its standardisation, costs and indications. In the meantime, it is advisable to discuss with patients the rarity of these variants, but also their implications, considering the costs of pharmacogenetic tests. Despite the recognised relevance of these genomic tests, treatment with fluoropyrimidines should not be substantially modified until a definitive recommendation based on the medical oncology community is generated taking into account all aspects of this molecular approach including access, cost and accuracy. Studies are needed to try to discover and describe possible new deleterious variants of the *DPYD* gene for South American populations. Thus, investments in testing and treatment protocols or dose adjustment can be better targeted, optimising expenses in a scarce resources scenario.

## Authors' contributions

All authors have made a significant contribution to this manuscript, have seen and approved the final manuscript and agree to its submission to the journal.

## Conflicts of interest

None.

## Funding

None to declare. This research did not receive any specific grant from funding agencies in the public, commercial or not-for-profit sectors.

## Warnings

The opinions expressed in the report presented are those of the authors and do not necessarily represent the official position of the institution to which they belong.

## Figures and Tables

**Table 1.** Activity value and functional status of strong evidence-based *DPYD* alleles.

Allele	Activity value	Allele functional status
Reference	1	Normal function
c.1905+1G>A (*2A)	0	No function
c.1129-5923C>G, c.1236G>A (HapB3)	0.5	Decreased function
c.2846A>T	0.5	Decreased function
c.1627A>G (*5)	1	Normal function
c.85T>C (*9A)	1	Normal function

Source: http://www.pharmgkb.org [32]

**Table 2.** Examples of diplotypes with respective AS and *DPYD* metaboliser predictors.

Variant 1	Variant 2	Sum of two lowest AS^a^	*DPYD* metaboliser
c.1905+1G>A (*2A)	c.1905+1G>A (*2A)	0	Poor
c.1905+1G>A (*2A)	c.1679T>G (*13)	0	Poor
c.1898delC (*3)	c.1679T>G (*13)	0	Poor
c.1679T>G (*13)	c.1679T>G (*13)	0	Poor
c.1905+1G>A (*2A)	c.2846A>T	0.5	Poor
c.1679T>G (*13)	c.557A>G	0.5	Poor
c.1679T>G (*13)	c.2846A>T	0.5	Poor
c.1679T>G (*13)	c.1129-5923C>G, c.1236G>A (HapB3)	0.5	Poor
c.1905+1G>A (*2A)	Any normal function variant	1	Intermediate
c.1679T>G (*13)	Any normal function variant	1	Intermediate
c.557A>G	c.557A>G	1	Intermediate
c.557A>G	c.2846A>T	1	Intermediate
c.557A>G	c.1129-5923C>G, c.1236G>A (HapB3)	1	Intermediate
c.2846A>T	c.2846A>T	1	Intermediate
c.1129-5923C>G, c.1236G>A (HapB3)	c.1129-5923C>G, c.1236G>A (HapB3)	1	Intermediate
c.557A>G	Any normal function variant	1.5	Intermediate
c.1129-5923C>G, c.1236G>A (HapB3)	Any normal function variant	1.5	Intermediate

Source: http://www.pharmgkb.org [32]

a AS, Activity score

**Table 3.** The allelic frequency of the four main *DPYD* variants according to the ABraOM repository.

SNP	dbSNP	c.	STAR nomenclature	MAF	Heterozygotes
2	rs115232898	c.557A>G	Non-described	0.256%	0.512%
4	rs75017182	c.1129-5923C>G; c.1129-5923C>A	hapB3	0.427%	0.854%
8	rs3918290	c.1905+1G>A; c.1905+1G>C;	DPYD*2A	0.128%	0.256%
9	rs67376798	c.2846A>T	Non-described	0.384%	0.768%

Source: https://abraom.ib.usp.br [34]

SNP, Single nucleotide polymorphism; dbSNP, databaseSNP; MAF, Minor allele frequencies

**Table 4.** Assignment of likely DPD phenotype based on genotype and respective toxicity risk.

Phenotype	Genotype	Activity Score	Risk of severe toxicity with fluoropyrimidine
*DPYD* normal metaboliser	There are two copies of normal function *DPYD* alleles. No selective dose adjustment is indicated for medications that are metabolised by DPD.	2	Low risk
*DPYD* intermediate metaboliser	There is one copy of a normal function allele and one copy of a decreased function allele of the *DPYD* gene. A fluoropyrimidine dose adjustment may be indicated.	1.5	High risk
*DPYD* intermediate metaboliser	The patient has either one copy of a normal function allele and one copy of a no function allele of the *DPYD* gene or two copies of decreased function alleles of the *DPYD* gene. A fluoropyrimidine dose adjustment may be indicated.	1	High risk
*DPYD* poor metaboliser	There is one copy of a decreased function allele and one copy of a no function allele of the* DPYD* gene. This patient may be at risk for adverse drug reactions to medications that are metabolised by DPD and a dose adjustment or alternative therapeutic agents to fluoropyrimidine may be indicated.	0.5	High risk
*DPYD* poor Metaboliser	There are two copies of no function alleles of the *DPYD* gene. An alternative therapeutic agent to fluoropyrimidine may be indicated.	0	High risk

Source: http://www.pharmgkb.org [32], Lunenburg *et al* [23] and Caudle *et al* [24]

**Table 5.** Incidence/prevalence of the most common cancers treated with fluoropyrimidines.

	Inca 2020^a^	Brazil^b^	South America^b^
Breast	66.280	299.542	536.725
Colorectal	41.010	133.376	256.895
Stomach	21.230	28.396	70.350
Oesophagus	11.390	10.991	16.452
Pancreas	-	10.260	21.150
Anus	-	7.628	12.273
Total	139.910	490.193	913.845

a Incidence by 100.000 habitants – Inca 2020 [49, 50]

b 5-year prevalence – Globocan 2020 [51]
